# SMO Inhibition Modulates Cellular Plasticity and Invasiveness in Colorectal Cancer

**DOI:** 10.3389/fphar.2017.00956

**Published:** 2018-02-02

**Authors:** Paolo Magistri, Cecilia Battistelli, Raffaele Strippoli, Niccolò Petrucciani, Teijo Pellinen, Lucia Rossi, Livia Mangogna, Paolo Aurello, Francesco D'Angelo, Marco Tripodi, Giovanni Ramacciato, Giuseppe Nigri

**Affiliations:** ^1^Department of Medical and Surgical Sciences and Translational Medicine, Sapienza—University of Rome, Rome, Italy; ^2^Molecular Genetics Section, Department of Cellular Biotechnology and Hematology, Sapienza—University of Rome, Rome, Italy; ^3^FIMM Institute for Molecular Medicine Finland, Helsinki, Finland

**Keywords:** colon cancer, hedgehog pathway, SMO inhibition, EMT, cell plasticity, cell invasiveness

## Abstract

**HIGHLIGHTS**

- Preliminary results of this work were presented at the 2016 Academic Surgical Congress, Jacksonville (FL), February 2–4 2016 (Original title: Selective Smo-Inhibition Interferes With Cellular Energetic Metabolism In Colorectal Cancer)
- This study was funded by “Sapienza—University of Rome” (Funds for young researchers) and “AIRC” (Italian Association for Cancer Research)
- Hedgehog inhibitor was kindly provided by Genentech, Inc.®.

Colon Cancer (CC) is the fourth most frequently diagnosed tumor and the second leading cause of death in the USA. Abnormalities of Hedgehog pathway have been demonstrated in several types of human cancers, however the role of Hedgehog (Hh) in CC remain controversial. In this study, we analyzed the association between increased mRNA expression of *GLI1* and *GLI2*, two Hh target genes, and CC survival and recurrence by gene expression microarray from a cohort of 382 CC patients. We found that patients with increased expression of *GLI1* showed a statistically significant reduction in survival. In order to demonstrate a causal role of Hh pathway activation in the pathogenesis of CC, we treated HCT 116, SW480 and SW620 CC cells lines with GDC-0449, a pharmacological inhibitor of Smoothened (SMO). Treatment with GDC-0449 markedly reduced expression of Hh target genes *GLI1, PTCH1, HIP1, MUC5AC*, thus indicating that this pathway is constitutively active in CC cell lines. Moreover, GDC-0449 partially reduced cell proliferation, which was associated with upregulation of p21 and downregulation of CycD1. Finally, treatment with the same drug reduced migration and three-dimensional invasion, which were associated with downregulation of Snail1, the EMT master gene, and with induction of the epithelial markers Cytokeratin-18 and E-cadherin. These results were confirmed by SMO genetic silencing. Notably, treatment with 5E1, a Sonic Hedgehog-specific mAb, markedly reduced the expression of Hedgehog target genes, as well as inhibited cell proliferation and mediated reversion toward an epithelial phenotype. This suggests the existence of a Hedgehog autocrine signaling loop affecting cell plasticity and fostering cell proliferation and migration/invasion in CC cell lines. These discoveries encourage future investigations to better characterize the role of Hedgehog in cellular plasticity and invasion during the different steps of CC pathogenesis.

## Introduction

In colon carcinoma (CC) as for other cancers, the understanding of cellular and molecular mechanisms involved in tumor progression is essential to provide the rationale for novel therapies. According to the 2016 NCCN Panel, CC is the fourth most frequently diagnosed tumor and the second leading cause of death in the USA. Incidence in Italy raised from 27.06% in 1970 to 52.65% in 2010 (Ciatto, 2007). Reactivation or alteration of molecular pathways that control cellular differentiation and proliferation play a role in the development and progression of both familiar and sporadic CC (Al-Sohaily et al., 2012; Bertrand et al., 2012). Numerous studies demonstrated that chemotherapy may be helpful both for primary and for metastatic tumors (Nappi et al., 2016). However, the persistence of cases of local recurrence in patients treated with current protocols impels to study the impact of novel pathways active in CC pathogenesis.

Hh pathway plays an important role in tissue development and organogenesis (Jiang and Hui, 2008; Rimkus et al., 2016). However, in mature organisms, Hh pathway remains selectively active, controlling cell proliferation and differentiation.

Hh ligands are expressed by various stromal and parenchymal tissues and act generally in a paracrine way by binding to the plasma membrane receptor Patched (PTCH1). Upon binding of Hh ligands to PTCH1, the G-protein–coupled receptor, Smoothened (SMO) is activated and promotes nuclear translocation of GLI family of zinc finger transcription factors. GLI activation induces the transcription of Hh target gene products, including ubiquitous genes such as GLI1, PTCH1, and Hh-interacting protein (HIP1) (Benvenuto et al., 2016; Rimkus et al., 2016). This canonical Hh signaling cascade plays a role in the normal gastrointestinal development, regulating the differentiation of normal intestinal villi and of the adjacent mesenchymal stromal cells. In particular, in the normal adult gastrointestinal tract, induction of the Hh pathway protects the differentiated epithelial cells of the villous surface at the top of the crypts of Lieberkuhn (the structural unit of the normal colon), counteracting the canonical Wnt signaling in the basal cells of the crypt. In the absence of Hh ligand, PTCH1 suppresses the activation of SMO.

Since Hh pathway is involved in the control of proliferation/differentiation status in many tissues, it is not surprising that Hh expression and activity are altered in tumors (Scales and de Sauvage, 2009).

Aberrant Hh signaling, which can be achieved by mutational inactivation of PTCH1, aberrant expression of its ligand, constitutive activation of SMO or gene amplification of GLI-associated transcription factors, has been implicated in the initiation and/or maintenance of different cancer types, including basal cell carcinoma (BCC), lung, and brain tumors and rhabdomyosarcoma (Gupta et al., 2010).

The involvement of Hh pathway in the pathogenesis of CC and the potential relevance for therapy has been already addressed by different studies. It has been demonstrated that Hh-GLI activity in epithelial tumor cells of CC is essential for tumor growth, recurrence and metastasis, and regulates the behavior of human CC stem cells *in vivo* (Varnat et al., 2009). However, a randomized phase II trial using a pharmacological inhibitor of Hh failed to show an incremental benefit respect to standard treatments in a population of previously untreated patients with metastatic CC (Berlin et al., 2013). In spite of this, recent research based on *in vitro* and *in vivo* experimental systems, led to controversial results (Gerling et al., 2016; Kangwan et al., 2016; Lee T. Y. et al., 2016; Wang et al., 2016).

The aim of the present study was to analyze whether pharmacological inhibition of Hh pathway, and specifically of SMO, impacts epithelial plasticity and invasiveness in different CC cell lines, and to explore the molecular mechanisms involved. Starting from our evidence correlating Hh pathway effectors (i.e., GLI1 and GLI2) with CC patients survival, we first treated CC cell lines with a pharmacological SMO inhibitor, GDC-0449, a small molecule already approved by the US Food and Drug Administration (FDA) for the treatment of advanced basal cell carcinoma (BCC) (Dijkgraaf et al., 2011; Takebe et al., 2015). In addition, we investigated the effects of SMO-specific genetic silencing as well as 5E1, a Shh-specific Monoclonal Antibody (mAb). Our results show that Hh pathway impacts epithelial/mesenchymal features and invasion capability of CC cell lines.

## Materials and methods

### Cells

Primary (HCT 116, SW480) and metastatic (SW620) human CC cell lines were grown in DMEM supplemented with 10% FBS (GIBCO® Life Technology, Monza, Italy) and antibiotics. These cellular lines were reported to express detectable levels of SMO and GLI1 (Sun et al., 2013; Sénicourt et al., 2016; Wang et al., 2017). MeT5A cells, a human mesothelium non-tumorigenic cell line were grown in M199 supplemented with 10% FBS (GIBCO® Life Technology, Monza, Italy) and antibiotics.

### RNA extraction, reverse transcription (RT), and real-time polymerase chain reaction (RT-qPCR)

RNA, extracted from cell cultures with ReliaPrep™ RNA Tissue Miniprep System (Promega, Madison, WI, USA), was reverse transcribed with iScriptTM c-DNA Synthesis Kit (Bio-Rad Laboratories, Inc., Hercules, CA, USA) according to the manufacturer's instructions. cDNAs were amplified by qPCR reaction using GoTaq® qPCR Master Mix (Promega, Madison, WI, USA). The specific primer pairs are listed in Table 1. Relative amounts, obtained with 2^−ΔCt^ method, were normalized with respect to the housekeeping gene L32. Statistical significance was determined with a *t*-test with GraphPad Prism version 5.0 (La Jolla, CA, USA). Differences were considered significant at *P* < 0.05 (^*^p < 0.05; ^**^p < 0.01; ^***^p < 0.001).

**Table 1:** List of specific primer pairs for qRT-PCR used in this study.

***Gene***	**Oligonucleotide sequence**
*L32 FW*	GGAGCGACTGCTACGGAAG
*L32 REV*	GATACTGTCCAAAAGGCTGGAA
*GLI1 FW*	GACGCCATGTTCAACTCGATG
*GLI1 REV*	CAGACAGTCCTTCTGTCCCCAC
*PTCH1 FW*	GAGCAGATTTCCAAGGGGAAGG
*PTCH1 REV*	ATGAGGAGGCCCACAACCAA
*HIP1 FW*	AGAACTGCAAAATGTGAGCCAG
*HIP1 REV*	CTGATCAAGAATACCTGCCCTG
*MUC5AC FW*	CCATTGCTATTATGCCCTGTGT
*MUC5AC REV*	TGGTGGACGGACAGTCACT
*CYC D1 FW*	CCTCTAAGATGAAGGAGACCA
*CYC D1 REV*	CACTTGAGCTTGTTCACCA
*P21 FW*	GAGGAGGCGCCATGTCAGAA
*P21 REV*	AGTCACCCTCCAGTGGTGTC
*SNAIL1 FW*	CACTATGCCGCGCTCTTTC
*SNAIL1 REV*	GCTGGAAGGTAAACTCTGGATTAGA
*CK18 FW*	CTGGAGACCGAGAACCGGA
*CK18 REV*	TCCGAGCCAGCTCGTCAT
*SMO FW*	TGAAGGCTGCACGAATGAGG
*SMO REV*	CTTGGGGTTGTCTGTCCGAA

### Antibodies and chemicals

Monoclonal antibody against E-cadherin was from BD (Becton-Dickinson Laboratories, Mountain View, CA). Monoclonal antibodies against Snail1 and against GLI1 were from Cell signaling (Danvers, MA). Monoclonal antibody against pan-cytokeratin was from Sigma (St Louis, MO). Polyclonal antibodies against SMO (N-19) and tubulin were from Santa Cruz biotechnology (Dallas, USA). GDC-0449, a Hedgehog/Smoothened pharmacological inhibitor, was a kind gift from Genentech (South San Francisco, CA). 5E1, Shh specific mAb was purchased by Developmental Studies Hybridoma Bank (Iowa City, Iowa, US) (Ericson et al., 1996).

### siRNA-mediated SMO knockdown

200 × 10^3^ cells were seeded on 12-well plates 24 h prior transfection. Cells were transfected with either 40 pmol ON-TARGETplus SMARTpool against human SMO (Dharmacon Ref#SO-2600349G) or the same amount of ON-TARGETplus NontargetingPool (Dharmacon Ref#D-001206-14-05) and 2 μl Lipofectamine® RNAiMAX Reagent from Thermo Fisher Scientific (Waltham, MA USA) in 200 μl Optimem from Gibco (Waltham, MA USA). 1 ml of supplemented medium per well was also added. Forty-eight hours after transfection, knockdown efficiency was determined by RT-PCR and western blot.

### Western blotting

Monolayers of HCT 116 cells were lysed in modified RIPA buffer containing: 50 mM Tris-HCl, pH 7.4; 1% NP-40; 0.1% SDS; 0.25% Nadeoxycholate; 150 mM NaCl; 1 mM EDTA; 1 mM EGTA; 1 mM PMSF; 1 μg/ml each of aprotinin, leupeptin, and pepstatin; and 25 mM NaF (all from Sigma). Equal amounts of proteins were resolved by SDS-PAGE. Proteins were transferred to nitrocellulose membranes, probed with antibodies and detected as in Battistelli et al. (2017).

#### Scratch assay

CC cell lines were pretreated with DMSO or 1 μM GDC-0449 for 24 h in culture medium until reaching 100% confluency. A scratch wound was created on the cell surface using a micropipette tip in low serum (0.5% FBS) culture medium to inhibit cell proliferation. Micrographs were taken at time 0 and 24 h after the scratch. Cell devoid areas at time 0 and 24 h after the scratch were quantified through ImageJ software (NIH, Bethesda, MD, USA). Three independent experiments for each cell line were performed

#### 3-Dimensional invasion assay and immunofluorescence

3-dimensional invasion assays were performed as in Strippoli et al. (2015). HCT 116 cells (1.5 × 105) were treated with 1 μM GDC-0449 or vehicle for 12 h and then seeded in triplicate in Ibidi 15-well chambers and allowed to attach for 3 h. 40% matrigel (40 μl) in serum-free medium was laid over the cells. After 1 h, 50 μl full medium containing 20% serum (and GDC-0449 or vehicle) was added and cells incubated for 72 h. Cells were fixed with 4% paraformaldehyde (PFA), permeabilized with 0.25% Triton in PBS, and stained for 12 h with rhodamine-phalloidin (to stain F-actin) and Hoechst (to stain nuclei) in PBS. Confocal images were captured with a Leica SP5 fitted with a 40 × oil objective. Maximum projection images consist of 30 individual images with a z-distance of 120 μm. Imaris image analysis software (Bitplane Scientific Software) was used to create a 3D view of the same cells and to quantify invading cells. Three independent experiments were performed.

### Statistical analysis

Survival analysis significance was based on Logrank Test. Statistical significance of experiments performed with cell lines was determined with a *t*-test using GraphPad Prism version 5.0 (La Jolla, CA, USA). Differences were considered significant at *P* < 0.05.

## Results

### GLI altered expression correlates with reduced survival in CC patients

To determine the clinical relevance of Hh-GLI pathway in CC, we examined the association between increased *GLI* mRNA expression and CC survival by exploring gene expression data microarray from a cohort of 382 CC patients in the cancer genome atlas (TCGA) database (Cerami et al., 2012; Gao et al., 2013).

**Figure 1:**
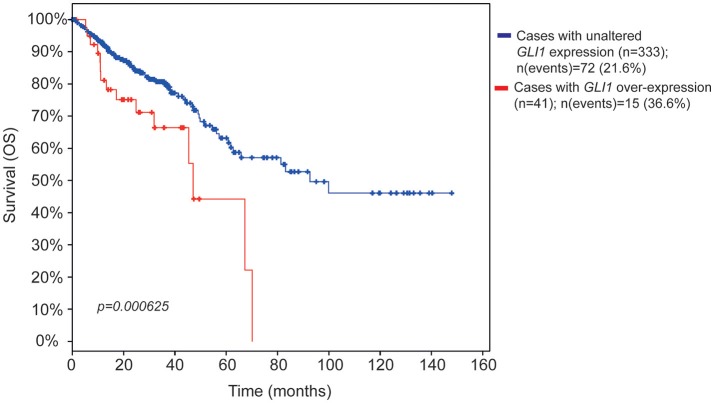
Kaplan-Meier survival estimate plots for increased *GLI* mRNA expression in CC patients. The survival estimate was analyzed in http://www.cbioportal.org (Cerami et al., 2012; Gao et al., 2013) and is based on Colorectal adenocarcinoma TCGA provisional dataset, which is generated by http://cancergenome.nih.gov/. The cases were set to include tumor samples with mRNA data (RNA Seq V2, *n* = 382 patients). The red line shows overall survival estimate for patients with increased *GLI1* expression (11% of the patients) as compared to patients with no alteration (blue line, the rest of the patients). Eight patients were missing in the survival analysis. The threshold for altered expression was set to z-score value = 1.2 The z-score value is used to define the cut-off for patient dichotomization in the TCGA datasets (see http://www.cbioportal.org/). The median months survival for the increased *GLI1* patient group and the reference group were 47.04 and 92.67, respectively. Survival analysis significance was based on Logrank Test. *P* < 0.05 was considered significant.

Notably, patients with increased *GLI1* expression showed a statistically significant reduction in overall survival (OS) (Figure 1) and a trend toward reduced disease-free survival (DFS) (Figure S1A). Patients with increased *GLI2* expression showed tendency to reduced survival (Figure S1B). These findings support a role for GLI1 and GLI2 in CC pathogenesis, and prompted us to investigate if pharmacological inhibition of Hh pathway could affect the proliferation or the invasive capacity of CC cell lines.

### GDC-0449 limits cellular proliferation of several CC cell lines

**Figure 2:**
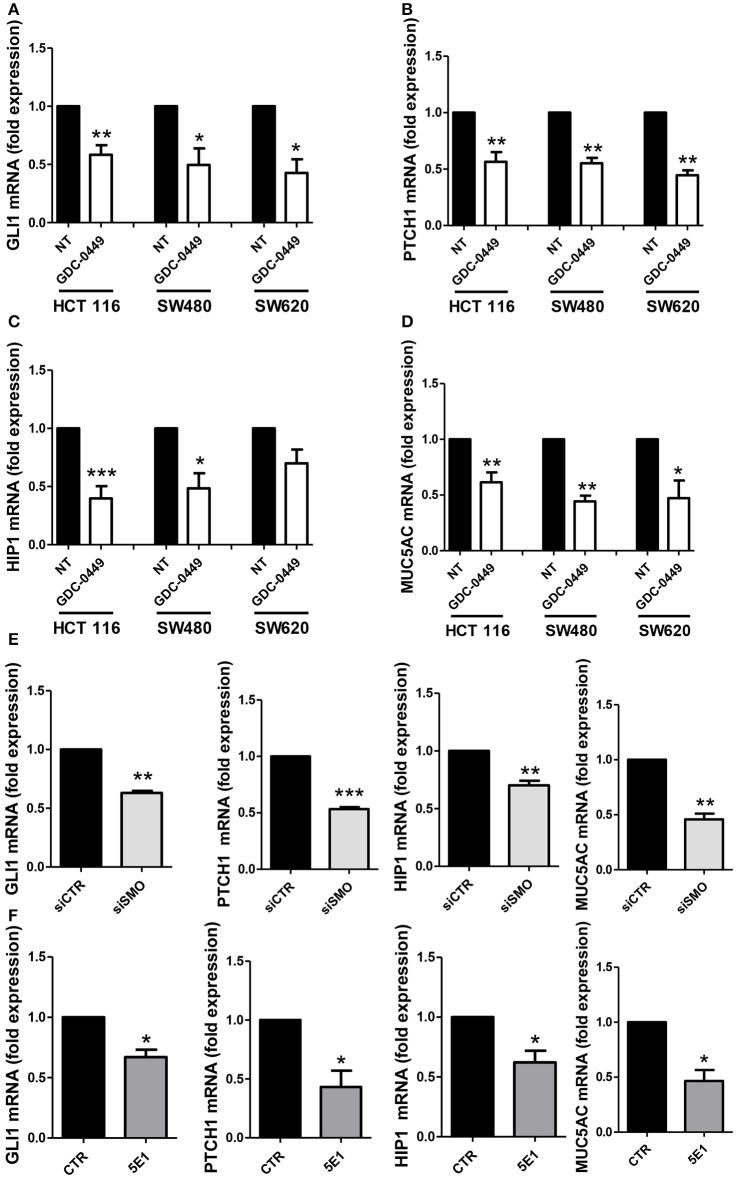
Effect of Hh inhibition on Hh pathway-induced genes. HCT 116, SW480 and SW620 were treated with DMSO vehicle (NT) or with 1 μM GDC-0449 for 24 h. Expression of *GLI1* **(A)**, *PTCH1* **(B)**, *HIP1* **(C)**, *MUC5AC* **(D)** was evaluated on total RNA by qRT-PCR. Bars represent means ± SEM of 7 experiment for HCT 116 cells, three experiments for SW480 and SW620 cells. Expression of *GLI1, PTCH1, HIP1, MUC5AC* in HCT 116 cells transfected with either control or specific SMO-targeting siRNAs **(E)**. Expression of *GLI1, PTCH1, HIP1, MUC5AC* in HCT 116 cells treated with either isotype-control mAb or 5E1 mAB **(F)**. Bars represent means ± SEM of three experiments. ^*^P < 0.05, ^**^P < 0.01, ^***^P < 0.001.

We analyzed whether treatment with GDC-0449 affects the expression of Hh pathway specific genes in CC lines. We exposed HCT 116, SW480, primary CC lines, and SW620, a metastatic CC cell line, to GDC-0449 for 24 h at a concentration (1 μM) that is compatible with cellular viability. As shown in Figures 2A–D, treatment with GDC-0449 significantly reduced the expression of *GLI1, PTCH1, HIP1, and MUC5AC*, four of the known Hh target genes, thus demonstrating that Hh pathway is constitutively active in all three cell lines. GLI1 protein expression upon treatment with GDC-0449 was shown in Figures S2A–C. Raw data of the original western blots shown in this study are displayed in Figures S5–S7.

Similar results were obtained by genetically silencing SMO, and by using 5E1, a mAb specifically binding to Shh (Figures 2E,F). The extent of SMO knockdown at mRNA level is shown in Figure S3A. Notably, the efficacy of 5E1 mAb, in the absence of exogenous stimulation, suggests the existence of an autocrine loop maintaining Hh pathway constitutively active in these cells.

Conversely in the MeT5A cell line, a non-tumorigenic mesothelium cell line, the same genes were found not responsive to the treatment with GDC-0449, therefore suggesting that these cells do not maintain a constitutively active SMO-GLI pathway (Figure S3B).

**Figure 3:**
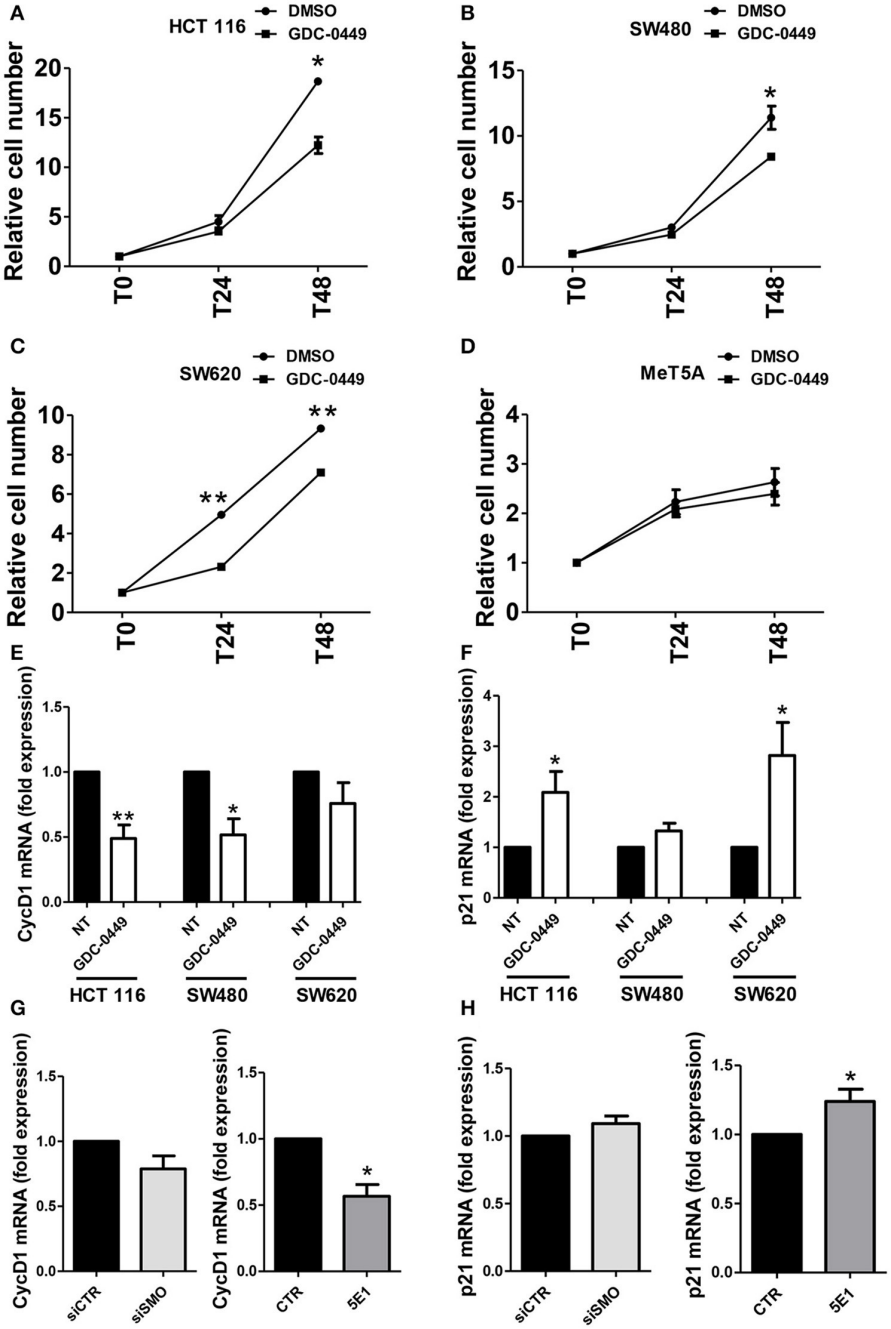
Effect of Hh inhibition on cell proliferation. **(A)** HCT 116, **(B)** SW480, **(C)** SW620 or **(D)** MeT5A cells were treated with vehicle (DMSO) or with GDC-0449 (1 μM) for 24–48 h. Cell proliferation was evaluated through microscopic count of living cells. Three independent experiments for each cell line were performed. Expression of *Cyclin D1* **(E)** and *p21* **(F)** was evaluated on total RNA by qRT-PCR. Bars represent means ± SEM of 7 experiment for HCT 116 cells, three experiments for SW480 and SW620 cells. **(G)** Expression of *Cyclin D1* mRNA was evaluated in HCT 116 cells transfected with either control or specific *SMO*-targeting siRNAs (left), or in HCT 116 cells treated with either isotype-control mAb or 5E1 mAb (10 μg/ml) (right). **(H)** Expression of *p21* mRNA was evaluated in HCT 116 cells transfected with either control or specific *SMO*-targeting siRNAs (left), or in HCT 116 cells treated with either isotype-control mAb or 5E1 mAb (10 μg/ml) (right). Bars represent means ± SEM of three independent experiments. ^*^P < 0.05, ^**^P < 0.01.

In order to evaluate the effect of SMO inhibition on cell proliferation, we exposed HCT 116, SW480, SW620, and MeT5A cells, to GDC-0449 for 24–48 h. At the concentration of 1 μM, GDC-0449 significantly inhibited cell proliferation in HCT 116, SW480, and SW620 while causing negligible effect in MeT5A cells (Figures 3A–D.) At the molecular level, treatment with GDC-0449 reduced the expression of cell cycle promoters (*Cyclin D1*) while inducing the expression of cell cycle inhibitors (*p21*) in all of the three CC lines analyzed (Figures 3E,F). Accordingly, both genetic silencing of *SMO* and treatment with 5EI mAb led to a reduction of *Cyclin D1* (Figure 3G), while increasing the expression of *p21* (Figure 3H). In contrast, no significative changes in the expression of these genes where detected when MeT5A were treated with GDC-0449 at the same concentrations (Figure S3C). These results demonstrate that activation of Hh pathway delivers proliferative signals in CC cell lines.

### GDC-0449 inhibits cell-directed motility and invasion and induces met

**Figure 4:**
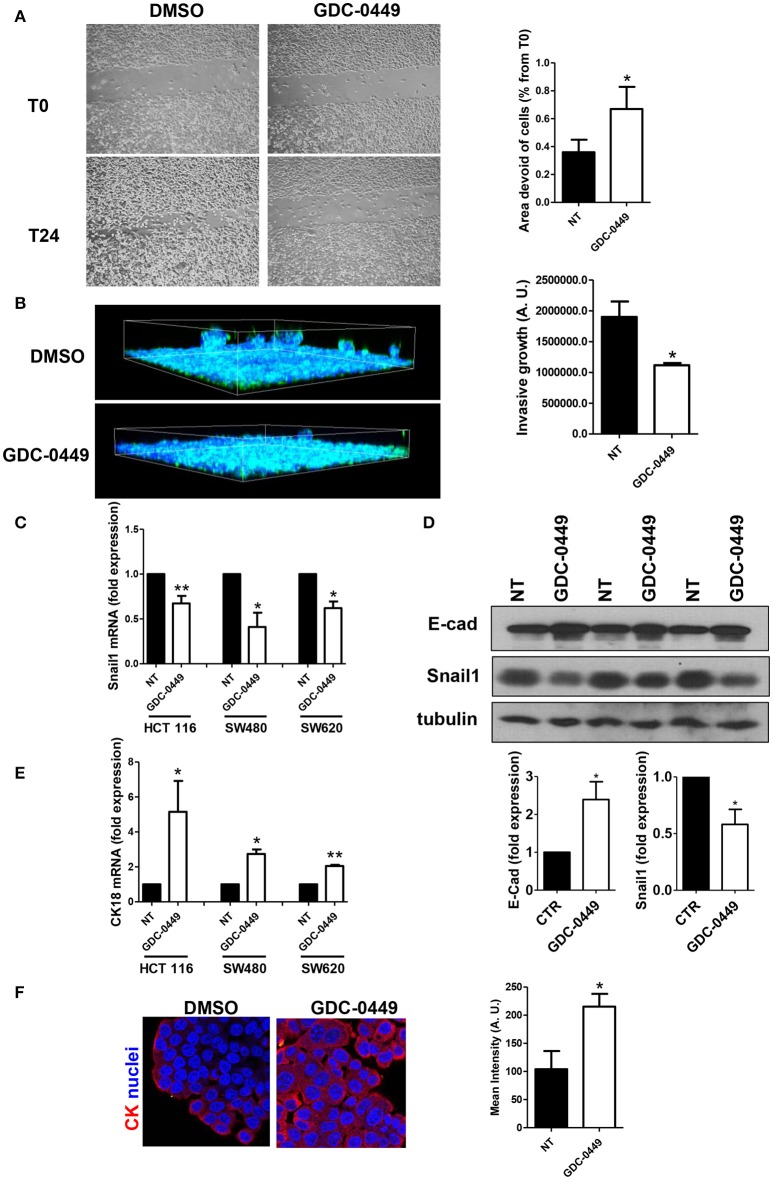
Effect of Hh inhibition on cell directed migration and on EMT-MET dynamics. **(A)** HCT 116 cells were treated with vehicle (DMSO) or with GDC-0449 (1 μM) in culture medium supplemented with 0.5% FCS. A scratch was performed and micrographs were taken 24 h after the scratch. Wound closure rate was quantified (right). Three independent experiments were performed. **(B)** HCT 116 cells were pretreated with GDC-0449 for 24 h and then overlaid with a Matrigel matrix. Invasion was monitored over 72 h. Three-dimensional invasion was enhanced by adding 20% FCS to the wells on top of Matrigel. Cells were stained with phalloidin (green) and Hoechst 33342 (cell nuclei, blue). Scale bar: 100 μm. 3 dimensional invasion rate was quantified (right). Three independent experiments were performed. **(C,D)** Effect of GDC-0449 on genes related to EMT-MET dynamics. **(C)** HCT 116, SW480, and SW620 cells were treated with GDC-0449 (1 μM) or DMSO for 24 h. Expression of *Snail1* mRNA was evaluated on total RNA by qRT-PCR. Bars represent means ± SEM of 7 experiment for HCT 116 cells, three experiments for SW480 and SW620 cells. **(D)** Expression of Snail1 and E–cadherin in cells treated as above by western blot on total protein lysates of HCT 116 cells treated as stated in the figure. Tubulin was used as a loading control. Three independent experiments were performed. Quantification of E-cadherin and Snail1 protein expression is shown below. **(E)** Expression of *Cytokeratin 18* (CK18) mRNA from total RNA of HCT 116 cells treated as in **(C)**. **(F)** Cytokeratin expression and localization in HCT 116 cells was evaluated through immunofluorescence analysis. Nuclei were stained in blue with Hoechst. Quantification of Cytokeratin mean intensity is shown on the right. Two independent experiments were performed. ^*^P < 0.05, ^**^P < 0.01.

We then analyzed whether GDC-0449 potentially inhibits tumor invasion. In order to analyze the effect of GDC-0449 in controlling cell directed motility, which is relevant for tumor invasion, we performed a scratch assay. Confluent HCT 116 monolayers were pre-treated with GDC-0449 for 24 h, a scratch was performed and the scratch closure was evaluated after 24 h. As shown in Figure 4A, treatment with GDC-0449 significantly limited scratch closure.

Similar results were obtained by using SW480 and SW620 cells (Figures S4A,B). By contrast GDC-0449 treatment had no effect on the Hh-irresponsive cell line MeT5A (Figure S4C).

Notably, the use of a 3-dimensional invasion assay on Matrigel matrices highlighted also a reduced invasive capacity of GDC-0449-treated HCT 116 cells (Figure 4B).

**Figure 5:**
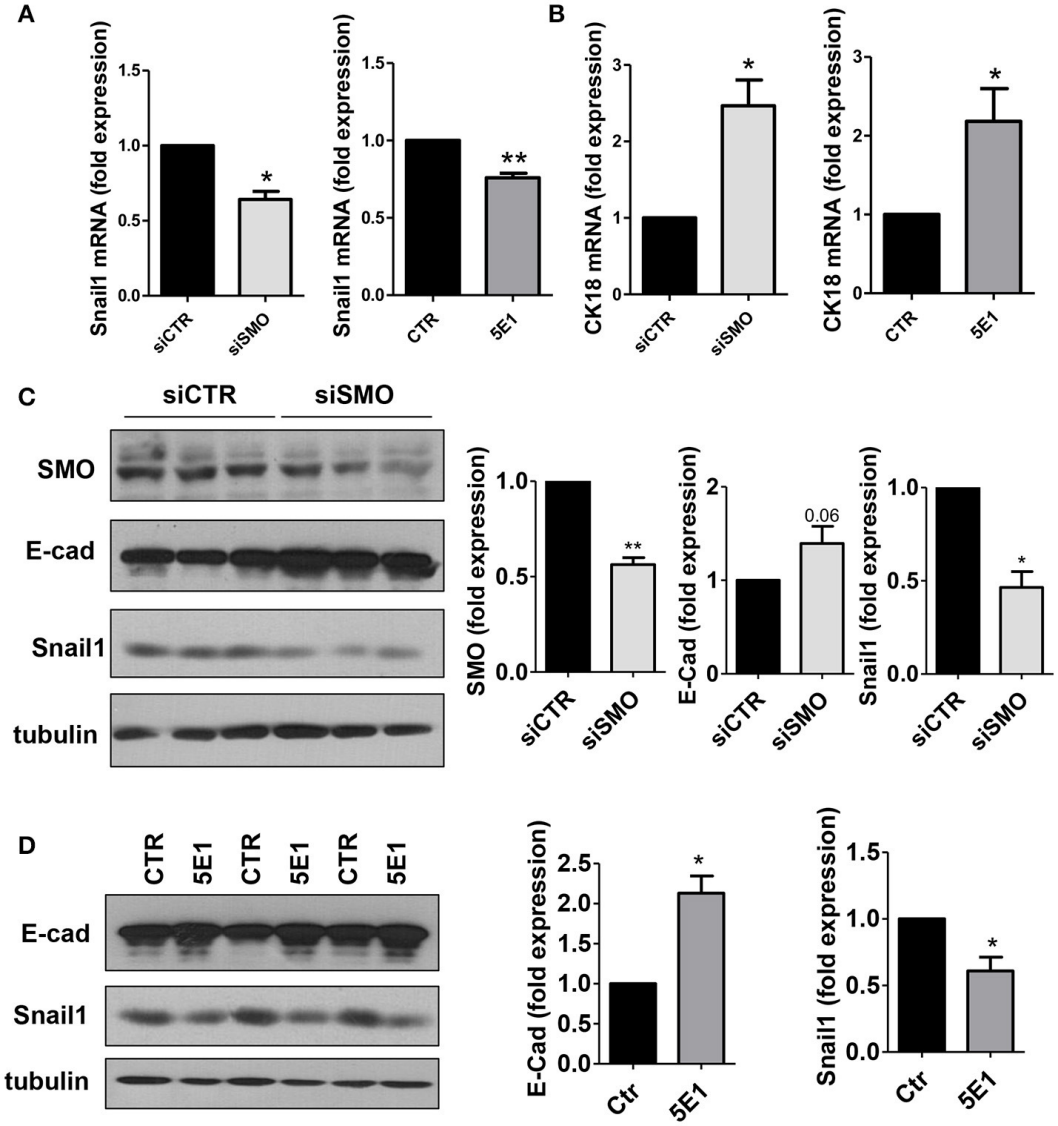
**(A)** *Snail1* and **(B)** *Cytokeratin 18* expression was evaluated on total RNA by qRT-PCR from HCT 116 cells transfected with either control or specific *SMO*-targeting siRNAs (left), or from cells treated with either isotype-control mAb or 5E1 mAb (10 μg/ml) (right) (*n* = 3). **(C)** Western blot showing expression of SMO, E-cadherin, Snail1 in HCT 116 cells transfected with either control or specific *SMO*-targeting siRNAs. Quantification of SMO, E-cadherin and Snail1 is shown on the left. (*n* = 3). **(D)** Western blot showing the expression of E-cadherin, Snail1 in HCT 116 cells treated with either isotype-control mAb or 5E1 mAb (10 μg/ml). Tubulin was used as loading control. E-cadherin and Snail1 quantifications are shown on the right. Bars represent means ± SEM of three independent experiments.^*^P < 0.05, ^**^P < 0.01.

At the molecular level, treatment with GDC-0449 markedly reduced the expression of Snail1, a main driver of EMT and tumor invasion, in all CC cell lines analyzed, while increasing the expression of the typical epithelial marker E-cadherin (Figures 4C,D). At the same time, Cytokeratin 18 (CK18) expression was increased both at mRNA and at protein level (Figures 4E,F). These results were confirmed by genetic silencing of SMO and by the use of 5E1 mAb (Figures 5A–D).

These data are in accordance with a data set from CC patients (Tables 2, 3), from the cancer genome atlas (TCGA) database (Cerami et al., 2012; Gao et al., 2013), where increased expression of Hh pathway genes (GLI1 and SMO) negatively correlates with protein expression of CDH1 (E-cadherin, epithelial marker) and positively correlate with CDH2 (N-Cadherin) and FN (Fibronectin), mesenchymal markers.

**Table 2:** Mean protein expression changes in CC patients with GLI1 mRNA overexpression.

**Gene**	**Mean protein expression**		**Stdev. of protein expression**		***p*-Value**
	**In GLI1 high group (*n* = 41)**	**In reference group (*n* = 333)**	**In GLI1 high group (*n* = 41)**	**In reference group (*n* = 333)**	
FASN	−0.5	0.1	0.59	0.6	0.000001046
FOXM1	−0.36	0.02	0.38	0.56	0.000001847
CCNB1	−0.77	0.02	0.9	0.78	0.000009887
CCNE1	−0.26	0.06	0.36	0.46	0.00001511
RPS6	−0.45	0.04	0.59	0.52	0.00002737
MSH6	−0.49	−0.02	0.58	0.6	0.00004738
ACACB_PS79	−0.34	0.02	0.48	0.46	0.0001271
ACACA_PS79	−0.34	0.02	0.48	0.46	0.0001271
SLC1A5	−0.63	−0.04	0.82	0.62	0.0001453
CASP7	−0.49	0.13	0.82	1.16	0.0001513
ACACA	−0.43	0	0.61	0.62	0.0002158
DIRAS3	0.23	0.06	0.23	0.33	0.0003006
FN1	0.75	0.15	0.87	0.78	0.0003219
PEA15	0.28	0.07	0.3	0.31	0.0003356
COL6A1	0.73	0.08	1	0.59	0.0004711
ARID1A	−0.12	0	0.19	0.27	0.0004835
CDH2	0.15	0.03	0.18	0.25	0.0005421
EIF4E	−0.15	0.04	0.29	0.29	0.000731
CHEK2	−0.29	0.09	0.62	0.44	0.0009215
RPS6KA1	−0.16	0.09	0.4	0.43	0.0009856
CCNE2	−0.07	0.05	0.19	0.24	0.001171
SMAD1	−0.13	0.02	0.23	0.23	0.001174
FOXO3_PS318	0.18	−0.03	0.36	0.24	0.001343
MSH2	−0.07	0.12	0.32	0.32	0.001649
EEF2	−0.23	0.01	0.4	0.41	0.001656
ASNS	−0.13	0.15	0.46	0.57	0.001962
HSPA1A	0.82	0.23	1.04	0.86	0.002055
ITGA2	−0.23	0.07	0.52	0.46	0.002136
PCNA	−0.21	0.01	0.4	0.37	0.002343
CTNNB1	−1.06	−0.32	1.34	1.01	0.002551
TGM2	0.22	0.06	0.28	0.31	0.002916
RB1	−0.06	0.12	0.32	0.32	0.003076
BCL2	0.22	0.02	0.35	0.45	0.003119
ACVRL1	0.3	0.04	0.49	0.37	0.00349
ERBB3	−0.27	0.05	0.59	0.61	0.004059
BAP1	−0.32	−0.08	0.46	0.48	0.004078
RPS6KB1	−0.3	−0.08	0.43	0.37	0.004689
ANXA1	0.34	0.03	0.6	0.59	0.005383
CDH1	−0.93	−0.3	1.24	1	0.005527
CDKN1A	0.2	0.03	0.33	0.36	0.00556
BRD4	−0.42	−0.11	0.63	0.5	0.006666
STK11	0.11	0.04	0.14	0.15	0.006734
SQSTM1	−0.31	−0.02	0.59	0.52	0.006856
EIF4EBP1	−0.13	0.04	0.35	0.34	0.007095
BAK1	0.12	0.04	0.17	0.21	0.007894
NRAS	0.17	0.08	0.19	0.23	0.008201
PGR	0.23	0.11	0.24	0.31	0.008854
CDK1_PY15	−0.23	0	0.48	0.31	0.008911
MRE11A	0.15	0.04	0.23	0.17	0.009363
AR	0.18	0.06	0.27	0.26	0.0104
WWTR1	0.13	0.01	0.27	0.27	0.0138
G6PD	0.15	0.04	0.24	0.31	0.0142
TP53BP1	−0.4	−0.05	0.78	0.72	0.0145
BECN1	0.13	0.02	0.24	0.22	0.015
RBM15	−0.54	−0.11	0.98	0.62	0.0159
TFRC	−0.35	0.02	0.84	0.85	0.0159
EIF4G1	−0.66	−0.27	0.88	0.93	0.0181
ESR1	0.19	0.07	0.28	0.36	0.0188
CLDN7	−0.63	−0.07	1.32	1.04	0.019
XRCC5	−0.27	−0.02	0.59	0.5	0.0194
TSC2	−0.32	−0.06	0.62	0.49	0.023
GAPDH	−0.39	−0.06	0.81	0.74	0.0289
MS4A1	0.12	0.04	0.22	0.18	0.029
KIT	0.28	−0.07	0.88	0.9	0.0297
FOXO3	0.13	0.05	0.19	0.24	0.03
GSK3B	−0.06	0.05	0.28	0.24	0.0306
GSK3A	−0.06	0.05	0.28	0.24	0.0306
CCND1	0.07	−0.01	0.21	0.23	0.0366
CDKN1B	0.21	0.09	0.32	0.34	0.0376
MAPK8_PT183	0.27	0.02	0.68	0.39	0.0383
RAB11B	0.14	0.03	0.29	0.24	0.041
RAB11A	0.14	0.03	0.29	0.24	0.041
CASP8	−0.04	0.14	0.45	0.79	0.0414
YAP1	0.29	0.14	0.42	0.37	0.0445

*Data was extracted from cBioPortal, Colorectal Adenocarcinoma (TCGA, Provisional), n(patients) = 382, z-score threshold = 1.2. The patient set was the same as in the survival analysis (see Figure 1). Protein measurements are based on antibody arrays (RPPA = Reverse Phase Protein Array), and the table shows only those proteins with significant p-value (p < 0.05). The z-score threshold used was the same as in the survival analysis (see Figure 1), where z-score 1.2 resulted in the best separation of patient groups into Gli1 mRNA overexpressing and non-overexpressing patients*.

**Table 3:** Mean protein expression changes in CC patients with SMO mRNA overexpression.

**Gene**	**Mean protein expression**		**Stdev. of protein expression**		***p*-Value**
	**In SMO high group (*n* = 54)**	**In reference group (*n* = 325)**	**In SMO high group (*n* = 54)**	**In reference group (*n* = 325)**	
RPS6KA1	−0.13	0.1	0.32	0.44	0.0001553
FN1	0.57	0.16	0.67	0.82	0.0005204
GAPDH	−0.42	−0.05	0.62	0.76	0.0006319
CLDN7	−0.61	−0.06	1.08	1.07	0.002821
CASP7	−0.36	0.13	0.98	1.15	0.003758
PTEN	−0.12	−0.03	0.21	0.22	0.006982
PEA15	0.21	0.08	0.29	0.32	0.008334
YAP1	0.32	0.13	0.43	0.36	0.0113
SYK	−0.21	0.03	0.59	0.54	0.0138
SLC1A5	−0.36	−0.07	0.76	0.65	0.0142
TFRC	−0.32	0.03	0.87	0.84	0.0172
VHL	−0.59	−0.18	1.04	1.21	0.0193
LCK	−0.06	0.09	0.37	0.47	0.0201
ERBB3	−0.17	0.04	0.51	0.62	0.0216
FOXO3_PS318	0.09	−0.02	0.29	0.26	0.0222
CTNNB1	−0.78	−0.34	1.15	1.05	0.0223
CDKN1B	0.2	0.08	0.32	0.34	0.0281
PECAM1	0.15	0.04	0.29	0.27	0.0282
MRE11A	0.11	0.04	0.2	0.17	0.0287
EEF2	−0.14	0	0.38	0.42	0.0304
RB1	0	0.11	0.3	0.33	0.0304
TSC2	−0.25	−0.07	0.52	0.51	0.0308
INPP4B	−0.17	0.01	0.5	0.63	0.0365
RBM15	−0.4	−0.12	0.82	0.65	0.0375
AR	0.16	0.06	0.31	0.25	0.0381
EGFR	−0.13	−0.05	0.21	0.25	0.0393
WWTR1	0.09	0.01	0.21	0.28	0.04
CDH1	−0.69	−0.32	1.11	1.03	0.0401
CDH2	0.1	0.03	0.2	0.25	0.0421
SMAD4	0.06	0	0.19	0.22	0.0448
SQSTM1	−0.2	−0.03	0.52	0.54	0.0451

*Data was extracted from cBioPortal, Colorectal Adenocarcinoma (TCGA, Provisional), n(patients) = 382, z-score threshold = 1.2. Protein measurements are based on antibody arrays (RPPA = Reverse Phase Protein Array), and the table shows only those proteins with significant p-value (p < 0.05). The z-score threshold 1.2 was selected based on optimal separation of patient groups into SMO mRNA overexpressing and non-overexpressing groups*.

Overall, these results demonstrate that in primary and metastatic CC cell lines, the functional blockade of autocrine Hh pathway leads to a reduction of cellular growth and invasion through a reacquisition of epithelial-like features. These results may be helpful to more completely understand the complex biology of CC in light of future therapeutic strategies.

## Discussion

The involvement of Hh in CC tumorigenesis is presently object of controversy. CC has a complex pathogenesis; during its progression, alterations of multiple genes and pathways are required. The observation that Hh pathway is tightly connected with other pathways that are often abnormally activated in CC, such as Wnt/β-catenin or Ras, strongly suggests its involvement in the multistep process of CC pathogenesis (Brechbiel et al., 2014). In fact, recent evidence indicates that Hh signaling may act in CC in autocrine, paracrine or cancer stem cell fashion (Scales and de Sauvage, 2009).

Notably, RNA Seq analysis in 382 patients highlighted a significative correlation between GLI1 increased expression and reduced survival in CC patients. These data support the results obtained by other studies (Xu et al., 2012), and prompted us to overcome the correlative analysis in pinpointing a mechanism; this attempt implied to inhibit Hh pathway in CC cell lines.

We first evaluated the effect of GDC-0449, a specific SMO inhibitor, on Hh signaling in three primary and metastatic CC cell lines. In all cells analyzed, treatment with GDC-0449 significantly reduced the expression of four known target genes (*GLI1, PTCH1, HIP1, MUC5AC)*, this implying a constitutive activation of Hh pathway. The activity of Hh pathway in CC cell lines has been already analyzed in other studies, with controversial results (Douard et al., 2006; Chatel et al., 2007; Alinger et al., 2009).

The observed discrepancies with some of these studies may be explained by the different cell lines analyzed (Alinger et al., 2009) or by the usage of semi-quantitative techniques (Chatel et al., 2007).

In our experimental system, treatment of all three lines with GDC-0449 also significantly reduced cell proliferation, whereas there was no effect in a not-responsive cell line used as a negative control.

At the molecular level, cell cycle inducers (Cyclin D1) were decreased, whereas cell cycle blockers (p21) were increased. Therefore, the use of GDC-0449 directly impacts cellular proliferation and potentially tumor growth. These results were confirmed by performing SMO genetic silencing and by using a 5E1 Shh-specific inhibitory mAb. The effect observed using 5E1 presupposes the existence of an autocrine activatory loop involving Shh-SMO-dependent canonical pathway activation. A previous report analyzing loss-of-function mutation in PTCH1 suggested a role of autocrine Hh signaling in colorectal tumorigenesis (Chung and Bunz, 2013). The autocrine loop observed by us in CC cell lines may complement other studies supporting a model in which paracrine Hh ligands released by CC cells take a role in instructing the stromal components of the tumor (Yauch et al., 2008).

Our results emphasize the role of canonical Hh signaling in CC cell lines, although we do not exclude that other pathways may play a role. Indeed, besides the canonical Hh-PTCH1-SMO-driven signals, ligand-independent pathways have been demonstrated (Merchant et al., 2004; Amakye et al., 2013). For instance, in tumors, GLI transcription may be decoupled from upstream Shh–PTCH1–SMO signaling being regulated by TGF-β and KRAS (Nolan-Stevaux et al., 2009).

Treatment with GDC-0449 also led to reduced cellular motility in both a 2D and a 3D migration assays. These functional data correlated with an increased expression of epithelial markers (E-cadherin, Cytokeratin-18) and with a significant downregulation of Snail1, a main effector of TGF-β pathway. With respect to cellular motility and Snail1 expression, our results appear equally provocative in the light of the fact that in several circumstances TGF-β facilitates tumor invasiveness through induction of epithelial-mesenchymal transition (EMT). EMT is a complex phenotypic conversion of epithelial cells leading to the acquisition of a highly motile phenotype and other mesenchymal traits, and is a key mechanism by which pre-malignant epithelial cells acquire a highly invasive phenotype that leads to metastatic spreading. The evidence of EMT in CC *in vivo* was firstly demonstrated by Brabletz (Brabletz et al., 2005). Subsequently, genomics studies identified EMT as a major program in CC (Loboda et al., 2011). Moreover, expression of EMT-related genes correlated with poorer survival in CC, but not in other cancers (Tan et al., 2014).

Cell migration and invasiveness are driven by actin cytoskeleton dynamic polarization, which is controlled by the members of the Rho GTPase superfamily Rho, Rac, and Cdc42 (Ridley, 2015). At the same time, EMT dynamics, as well as the acquisition of stem-like phenotypes are characterized by selective activation of Rho GTPases (Yoon et al., 2017). Interestingly, recent studies link Hh pathway to Rho GTPases activation, providing potential molecular mechanisms for our observations (Peng et al., 2017).

Hh pathway has been shown to intersect and cooperate with other pathways by regulating the differentiation/proliferation status of the tissue. In the intestinal mucosa, Bone Morphogenetic Protein (BMP) and Hh pathways are preferentially activated in differentiated layers, whereas Notch and WNT pathways are activated in the basal cells of the crypt (Bertrand et al., 2012). Hh controls the expression of the Notch ligand Jagged2. Moreover, cross talks between TGF-β-induced pathways and Hh have been demonstrated (Perrot et al., 2013). There is evidence of an interplay between Hh and TGF-β pathways in both normal and malignant tissues. In particular, TGF-β may induce GLI1 expression in a GLI2-dependent manner independently from SMO in breast cancer (Hu et al., 2008). Furthermore, pharmacologic blockade of TGF-β signaling leads to a reduction of GLI1 expression in cyclopamine-resistant pancreatic adenocarcinoma (Dennler et al., 2007).

We hypothesize that in our cells Snail1 downregulation, mediated by Hh-pathway inhibition, contributes to the stabilization of an epithelial phenotype (underlined by the increase in E-cadherin and Cytokeratin 18 expression). Besides the well-known effect on tumor invasion, Snail1 downregulation may be effective in limiting chemoresistance, as recently demonstrated in CC (Lee T. Y. et al., 2016). Accordingly, Hh pathway inhibition by GDC-0449 was demonstrated to reduce the expression of a mesenchymal marker and to restore sensitivity to 5-fluorouracil in a 5-fluorouracil-resistant CC cell line (Liu et al., 2015).

While the role of Hh pathway in EMT induction in CC has already been demonstrated by Ruiz i Altaba and other groups (Varnat et al., 2009; Wang et al., 2012; Liu et al., 2015), our study firstly described the maintenance of an invasive “EMT like state” by an autocrine secretion of Hh ligands in CC cell lines. This cellular mechanism may play a role in promoting cellular invasion and metastatic spreading of CC to the liver, consistently with our previous findings on tumor-stroma interactions in liver neoplasms (Magistri et al., 2013). The impact of Hh pathway on CC tumorigenesis has been recently questioned in mouse models (Gerling et al., 2016; Lee J. J. et al., 2016). Using different experimental systems such as the AOM/DSS model of colitis-associated CC, it was demonstrated that Hh signaling is downregulated in CC, and that Hh pathway inhibition may intensify colon inflammation (colitis) in mice, thus promoting tumorigenesis.

Conversely, other recent studies linked the same pathway activation to pro-tumor effects and to the acquisition of a cancer stem-like behavior (Kangwan et al., 2016; Wang et al., 2016).

Finally, it has been demonstrated that Hh active pathway may hamper tumor immunosurveillance (for example downregulating MHC1 expression in epithelial tumor cells), inhibiting the immune response against the tumor (Otsuka et al., 2015; Hanna and Shevde, 2016). Therefore, the interpretation of *in vivo* models is so far extremely challenging.

In conclusion, our *in vitro* study established a causal link between constitutive Hh activity and the acquisition of pro-invasive, mesenchymal-like properties in CC cell lines.

When looking for a possible translational relevance of these discoveries, one may hypothesize that subsets of colorectal tumors endowed with abnormal Hh signaling due to mutations, such as a recently reported PTCH1 loss-of-function mutation (Chung and Bunz, 2013), that may not have been represented in the cohorts previously treated with Hh inhibitors (Berlin et al., 2013), may benefit from drugs inhibiting Hh activity such as GDC-0449.

## Author contributions

PM, CB, and RS: equally contributed to the final manuscript and are responsible for study design, lab experiments, and manuscript drafting; GN, MT, and GR: supervised all the study process and revised the final manuscript; NP, LR, and FD: are responsible for literature review and data analysis; PA and LM: are responsible for lab experiments duplicates and statistical analysis. TP is responsible for the analysis of TCGA database.

### Conflict of interest statement

The authors declare that the research was conducted in the absence of any commercial or financial relationships that could be construed as a potential conflict of interest.

## References

[B1] Alinger B. Kiesslich T. Datz C. Aberger F. Strasser F. Berr F.. (2009). Hedgehog signaling is involved in differentiation of normal colonic tissue rather than in tumor proliferation. Virchows Archiv. 454, 369–379. 10.1007/s00428-009-0753-7 19280222

[B2] Al-Sohaily S. Biankin A. Leong R. Kohonen-Corish M. Warusavitarne J. (2012). Molecular pathways in colorectal cancer. J. Gastroenterol. Hepatol. 27, 1423–1431. 10.1111/j.1440-1746.2012.07200.x 22694276

[B3] Amakye D. Jagani Z. Dorsch M. (2013). Unraveling the therapeutic potential of the Hedgehog pathway in cancer. Nat. Med. 19, 1410–1422. 10.1038/nm.3389 24202394

[B4] Battistelli C. Cicchini C. Santangelo L. Tramontano A. Grassi L. Gonzalez F. J.. (2017). The Snail repressor recruits EZH2 to specific genomic sites through the enrollment of the lncRNA HOTAIR in epithelial-to-mesenchymal transition. Oncogene 36, 942–955. 10.1038/onc.2016.260 27452518 PMC5318668

[B5] Benvenuto M. Masuelli L. De Smaele E. Fantini M. Mattera R. Cucchi D.. (2016). *In vitro* and *in vivo* inhibition of breast cancer cell growth by targeting the Hedgehog/GLI pathway with SMO (GDC-0449) or GLI (GANT-61) inhibitors. Oncotarget 7, 9250–9270. 10.18632/oncotarget.7062 26843616 PMC4891038

[B6] Berlin J. Bendell J. C. Hart L. L. Firdaus I. Gore I. Hermann R. C.. (2013). A randomized phase II trial of vismodegib versus placebo with FOLFOX or FOLFIRI and bevacizumab in patients with previously untreated metastatic colorectal cancer. Clin. Cancer Res. 19, 258–267. 10.1158/1078-0432.CCR-12-1800 23082002

[B7] Bertrand F. E. Angus C. W. Partis W. J. Sigounas G. (2012). Developmental pathways in colon cancer: crosstalk between WNT, BMP, Hedgehog and Notch. Cell Cycle 11, 4344–4351. 10.4161/cc.22134 23032367 PMC3552917

[B8] Brabletz T. Hlubek F. Spaderna S. Schmalhofer O. Hiendlmeyer E. Jung A.. (2005). Invasion and metastasis in colorectal cancer: epithelial-mesenchymal transition, mesenchymal-epithelial transition, stem cells and beta-catenin. Cells Tissues Organs 179, 56–65. 10.1159/000084509 15942193

[B9] Brechbiel J. Miller-Moslin K. Adjei A. A. (2014). Crosstalk between hedgehog and other signaling pathways as a basis for combination therapies in cancer. Cancer Treat. Rev. 40, 750–759. 10.1016/j.ctrv.2014.02.003 24613036

[B10] Cerami E. Gao J. Dogrusoz U. Gross B. E. Sumer S. O. Aksoy B. A.. (2012). The cBio cancer genomics portal: an open platform for exploring multidimensional cancer genomics data. Cancer Discov. 2, 401–404. 10.1158/2159-8290.CD-12-0095 22588877 PMC3956037

[B11] Chatel G. Ganeff C. Boussif N. Delacroix L. Briquet A. Nolens G.. (2007). Hedgehog signaling pathway is inactive in colorectal cancer cell lines. Int. J. Cancer 121, 2622–2627. 10.1002/ijc.22998 17683069

[B12] Chung J. H. Bunz F. (2013). A loss-of-function mutation in PTCH1 suggests a role for autocrine hedgehog signaling in colorectal tumorigenesis. Oncotarget 4, 2208–2211. 10.18632/oncotarget.1651 24368541 PMC3926820

[B13] Ciatto S. (2007). Current cancer profiles of the Italian regions: should cancer incidence be monitored at a national level? Tumori 93, 529–531. 18338483 10.1177/030089160709300601

[B14] Dennler S. André J. Alexaki I. Li A. Magnaldo T. ten Dijke P.. (2007). Induction of sonic hedgehog mediators by transforming growth factor-beta: Smad3-dependent activation of Gli2 and Gli1 expression *in vitro* and *in vivo*. Cancer Res. 67, 6981–6986. 10.1158/0008-5472.can-07-0491 17638910

[B15] Dijkgraaf G. J. Alicke B. Weinmann L. Januario T. West K. Modrusan Z.. (2011). Small molecule inhibition of GDC-0449 refractory smoothened mutants and downstream mechanisms of drug resistance. Cancer Res. 71, 435–444. 10.1158/0008-5472.CAN-10-2876 21123452

[B16] Douard R. Moutereau S. Pernet P. Chimingqi M. Allory Y. Manivet P.. (2006). Sonic Hedgehog-dependent proliferation in a series of patients with colorectal cancer. Surgery 139, 665–670. 10.1016/j.surg.2005.10.012 16701100

[B17] Ericson J. Morton S. Kawakami A. Roelink H. Jessell T. M. (1996). Two critical periods of Sonic Hedgehog signaling required for the specification of motor neuron identity. Cell 87, 661–673. 8929535 10.1016/s0092-8674(00)81386-0

[B18] Gao J. Aksoy B. A. Dogrusoz U. Dresdner G. Gross B. Sumer S. O.. (2013). Integrative analysis of complex cancer genomics and clinical profiles using the cBioPortal. Sci. Signal. 6:pl1. 10.1126/scisignal.2004088 23550210 PMC4160307

[B19] Gerling M. Buller N. V. Kirn L. M. Joost S. Frings O. Englert B. (2016). Stromal Hedgehog signalling is downregulated in colon cancer and its restoration restrains tumour growth. Nat. Commun. 7:12321 10.1038/ncomms12321 27492255 PMC4980446

[B20] Gupta S. Takebe N. Lorusso P. (2010). Targeting the Hedgehog pathway in cancer. Ther. Adv. Med. Oncol. 2, 237–250. 10.1177/1758834010366430 21789137 PMC3126020

[B21] Hanna A. Shevde L. A. (2016). Hedgehog signaling: modulation of cancer properies and tumor mircroenvironment. Mol. Cancer 15, 24. 10.1186/s12943-016-0509-3 26988232 PMC4797362

[B22] Hu M. Yao J. Carroll D. K. Weremowicz S. Chen H. Carrasco D.. (2008). Regulation of *in situ* to invasive breast carcinoma transition. Cancer Cell 13, 394–406. 10.1016/j.ccr.2008.03.007 18455123 PMC3705908

[B23] Jiang J. Hui C. C. (2008). Hedgehog signaling in development and cancer. Dev. Cell 15, 801–812. 10.1016/j.devcel.2008.11.010 19081070 PMC6443374

[B24] Kangwan N. Kim Y. J. Han Y. M. Jeong M. Park J. M. Go E. J.. (2016). Sonic hedgehog inhibitors prevent colitis-associated cancer via orchestrated mechanisms of IL-6/gp130 inhibition, 15-PGDH induction, Bcl-2 abrogation, and tumorsphere inhibition. Oncotarget 7, 7667–7682. 10.18632/oncotarget.6765 26716648 PMC4884946

[B25] Lee T. Y. Liu C. L. Chang Y. C. Nieh S. Lin Y. S. Jao S. W.. (2016). Increased chemoresistance via snail-raf kinase inhibitor protein signaling in colorectal cancer in response to a nicotine derivative. Oncotarget 7, 23512–23520. 10.18632/oncotarget.8049 26992205 PMC5029643

[B26] Lee J. J. Rothenberg M. E. Seeley E. S. Zimdahl B. Kawano S. Lu W. J.. (2016). Control of inflammation by stromal Hedgehog pathway activation restrains colitis. Proc. Natl. Acad. Sci. U.S.A. 113, E7545–E7553. 10.1073/pnas.1616447113 27815529 PMC5127312

[B27] Liu Y. Du F. Zhao Q. Jin J. Ma X. Li H. (2015). Acquisition of 5-fluorouracil resistance induces epithelial-mesenchymal transitions through the Hedgehog signaling pathway in HCT-8 colon cancer cells. Oncol. Lett. 9, 2675–2679. 10.3892/ol.2015.3136 26137127 PMC4473303

[B28] Loboda A. Nebozhyn M. V. Watters J. W. Buser C. A. Shaw P. M. Huang P. S.. (2011). EMT is the dominant program in human colon cancer. BMC Med. Genom. 4:9. 10.1186/1755-8794-4-9 21251323 PMC3032646

[B29] Magistri P. Leonard S. Y. Tang C. M. Chan J. C. Lee T. E. Sicklick J. K. (2013). The glypican 3 hepatocellular carcinoma marker regulates human hepatic stellate cells via Hedgehog signaling. J. Surg. Res. 187, 377–385. 10.1016/j.jss.2013.12.010 24439425

[B30] Merchant M. Vajdos F. F. Ultsch M. Maun H. R. Wendt U. Cannon J.. (2004). Suppressor of fused regulates Gli activity through a dual binding mechanism. Mol. Cell. Biol. 24, 8627–8641. 10.1128/MCB.24.19.8627-8641.2004 15367681 PMC516763

[B31] Nappi A. Nasti G. Romano C. Cassata A. Silvestro L. Ottaiano A. (2016). Multimodal treatment of recurrent colorectal cancer. World Cancer Res. J. 3:e719.

[B32] Nolan-Stevaux O. Lau J. Truitt M. L. Chu G. C. Hebrok M. Fernández-Zapico M. E.. (2009). GLI1 is regulated through Smoothened-independent mechanisms in neoplastic pancreatic ducts and mediates PDAC cell survival and transformation. Genes Dev. 23, 24–36. 10.1101/gad.1753809 19136624 PMC2632164

[B33] Otsuka A. Dreier J. Cheng P. F. Nägeli M. Lehmann H. Felderer L.. (2015). Hedgehog pathway inhibitors promote adaptive immune responses in basal cell carcinoma. Clin. Cancer Res. 21, 1289–1297. 10.1158/1078-0432.CCR-14-2110 25593302

[B34] Peng W. X. Zhu S. L. Zhang B. Y. Shi Y. M. Feng X. X. Liu F.. (2017). Smoothened regulates migration of fibroblast-like synoviocytes in rheumatoid arthritis via activation of Rho GTPase signaling. Front. Immunol. 8:159. 10.3389/fimmu.2017.00159 28261216 PMC5309251

[B35] Perrot C. Y. Javelaud D. Mauviel A. (2013). Overlapping activities of TGF-beta and Hedgehog signaling in cancer: therapeutic targets for cancer treatment. Pharmacol. Ther. 137, 183–199. 10.1016/j.pharmthera.2012.10.002 23063491

[B36] Ridley A. J. (2015). Rho GTPase signalling in cell migration. Curr. Opin. Cell Biol. 36, 103–112. 10.1016/j.ceb.2015.08.005 26363959 PMC4728192

[B37] Rimkus T. K. Carpenter R. L. Qasem S. Chan M. Lo H. W. (2016). Targeting the sonic hedgehog signaling pathway: review of smoothened and GLI inhibitors. Cancers 8:22. 10.3390/cancers8020022 26891329 PMC4773745

[B38] Scales S. J. de Sauvage F. J. (2009). Mechanisms of Hedgehog pathway activation in cancer and implications for therapy. Trends Pharmacol. Sci. 30, 303–312. 10.1016/j.tips.2009.03.007 19443052

[B39] Sénicourt B. Boudjadi S. Carrier J. C. Beaulieu J. F. (2016). Neoexpression of a functional primary cilium in colorectal cancer cells. Heliyon 2:e00109. 10.1016/j.heliyon.2016.e00109 27441280 PMC4946219

[B40] Strippoli R. Loureiro J. Moreno V. Benedicto I. Perez Lozano M. L. Barreiro O. (2015). Caveolin-1 deficiency induces a MEK-ERK1/2-Snail-1-dependent epithelial-mesenchymal transition and fibrosis during peritoneal dialysis. EMBO Mol. Med. 7, 102–123. 10.15252/emmm.201404127 25550395 PMC4309670

[B41] Sun K. Deng H. J. Lei S. T. Dong J. Q. Li G. X. (2013). miRNA-338-3p suppresses cell growth of human colorectal carcinoma by targeting smoothened. World J. Gastroenterol. 19, 2197–2207. 10.3748/wjg.v19.i14.2197 23599646 PMC3627884

[B42] Takebe N. Miele L. Harris P. J. Jeong W. Bando H. Kahn M.. (2015). Targeting notch, Hedgehog, and Wnt pathways in cancer stem cells: clinical update. Nat. Rev. Clin. Oncol. 12, 445–464. 10.1038/nrclinonc.2015.61 25850553 PMC4520755

[B43] Tan T. Z. Miow Q. H. Miki Y. Noda T. Mori S. Thiery J. P.. (2014). Epithelial-mesenchymal transition spectrum quantification and its efficacy in deciphering survival and drug responses of cancer patients. EMBO Mol. Med. 6, 1279–1293. 10.15252/emmm.201404208 25214461 PMC4287932

[B44] Varnat F. Duquet A. Malerba M. Zbinden M. Mas C. Gervaz P.. (2009). Human colon cancer epithelial cells harbour active HEDGEHOG-GLI signalling that is essential for tumour growth, recurrence, metastasis and stem cell survival and expansion. EMBO Mol. Med. 1, 338–351. 10.1002/emmm.200900039 20049737 PMC3378144

[B45] Wang D. Hu G. Du Y. Zhang C. Lu Q. Lv N.. (2017). Aberrant activation of hedgehog signaling promotes cell proliferation via the transcriptional activation of forkhead Box M1 in colorectal cancer cells. J. Exp. Clin. Cancer Res. 36:23. 10.1186/s13046-017-0491-7 28148279 PMC5288899

[B46] Wang R. Wei J. Zhang S. Wu X. Guo J. Liu M.. (2016). Peroxiredoxin 2 is essential for maintaining cancer stem cell-like phenotype through activation of Hedgehog signaling pathway in colon cancer. Oncotarget 7, 86816–86828. 10.18632/oncotarget.13559 27894099 PMC5349956

[B47] Wang T. P. Hsu S. H. Feng H. C. Huang R. F. (2012). Folate deprivation enhances invasiveness of human colon cancer cells mediated by activation of sonic hedgehog signaling through promoter hypomethylation and cross action with transcription nuclear factor-kappa B pathway. Carcinogenesis 33, 1158–1168. 10.1093/carcin/bgs138 22461522

[B48] Xu M. Li X. Liu T. Leng A. Zhang G. (2012). Prognostic value of hedgehog signaling pathway in patients with colon cancer. Med. Oncol. 29, 1010–1016. 10.1007/s12032-011-9899-7 21424326

[B49] Yauch R. L. Gould S. E. Scales S. J. Tang T. Tian H. Ahn C. P.. (2008). A paracrine requirement for hedgehog signalling in cancer. Nature 455, 406–410. 10.1038/nature07275 18754008

[B50] Yoon C. Cho S. J. Chang K. K. Park D. J. Ryeom S. Yoon S. S. (2017). Role of Rac1 pathway in epithelial-to-mesenchymal transition and cancer stem-like cell phenotypes in gastric adenocarcinoma. Mol. Cancer Res. 15, 1106–1116. 10.1158/1541-7786.MCR-17-0053 28461325 PMC5540756

