# Supplementary figures and images for: SMO Inhibition Modulates Cellular Plasticity and Invasiveness in Colorectal Cancer
Source: Front Pharmacol. 2018 Feb 2;8:956. doi: 10.3389/fphar.2017.00956 (PMC5801594; doi:10.3389/fphar.2017.00956)

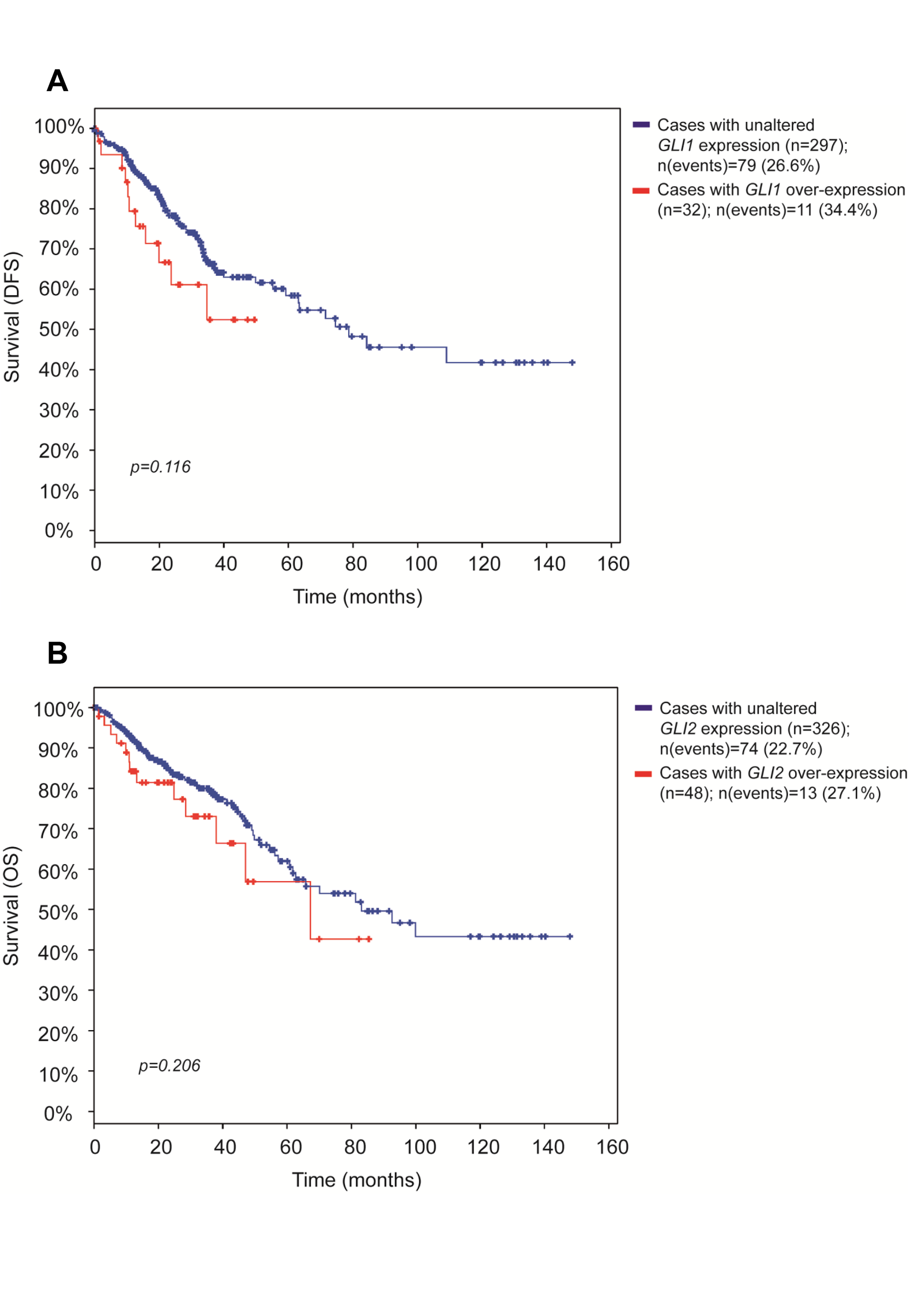

Supplement: Supplementary file 1 [file Image1.TIF]

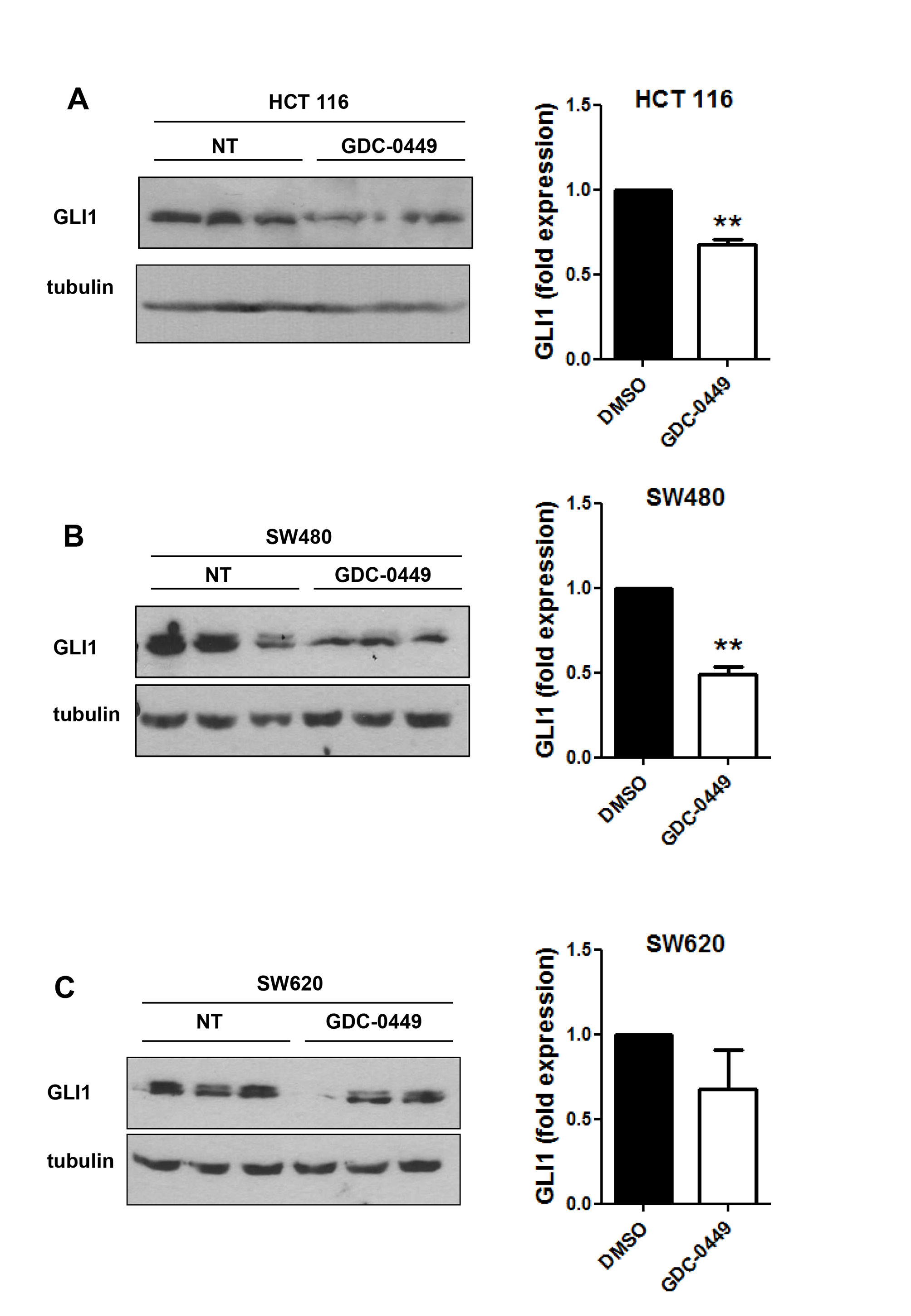

Supplement: Supplementary file 2 [file Image2.TIF]

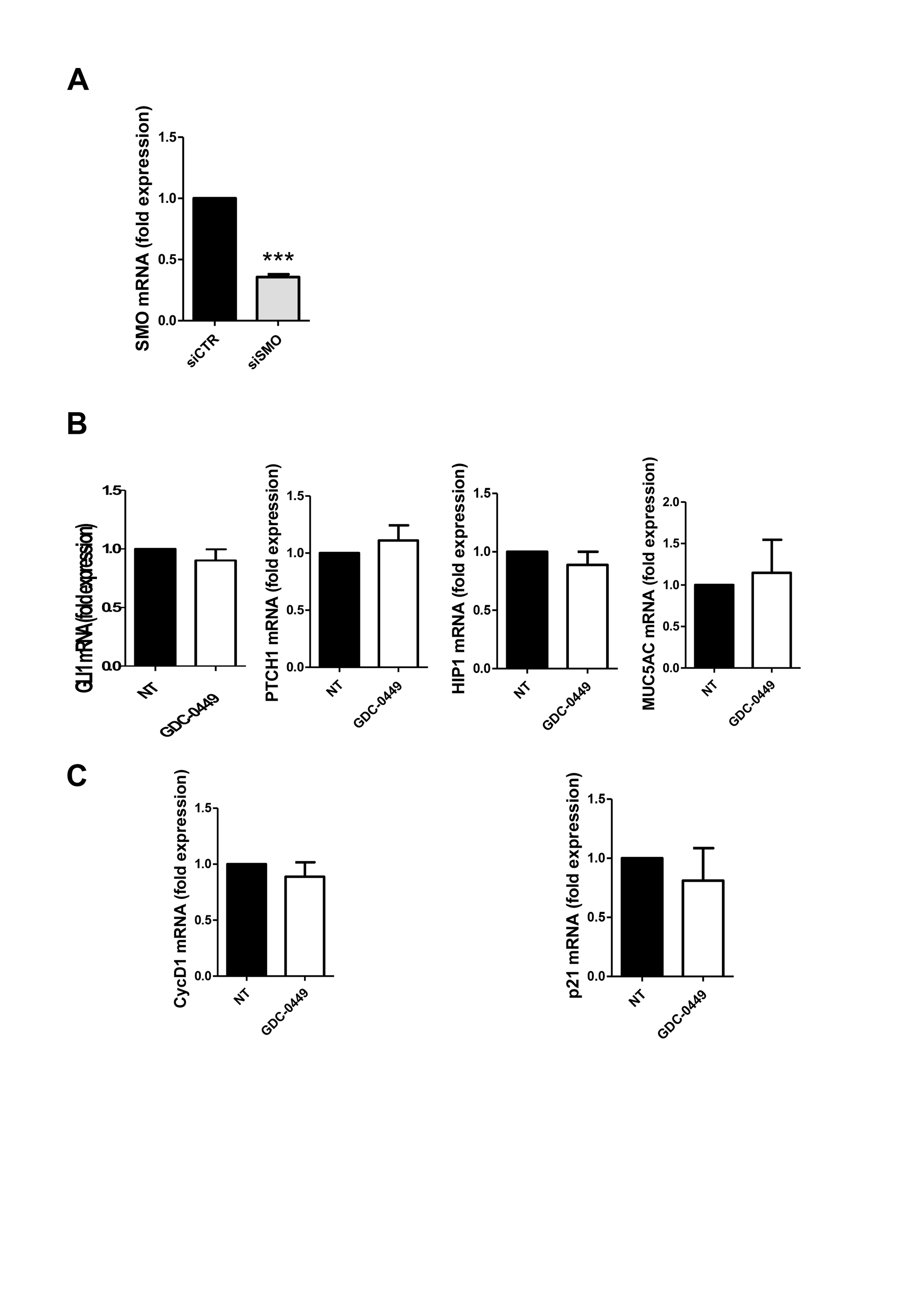

Supplement: Supplementary file 3 [file Image3.TIF]

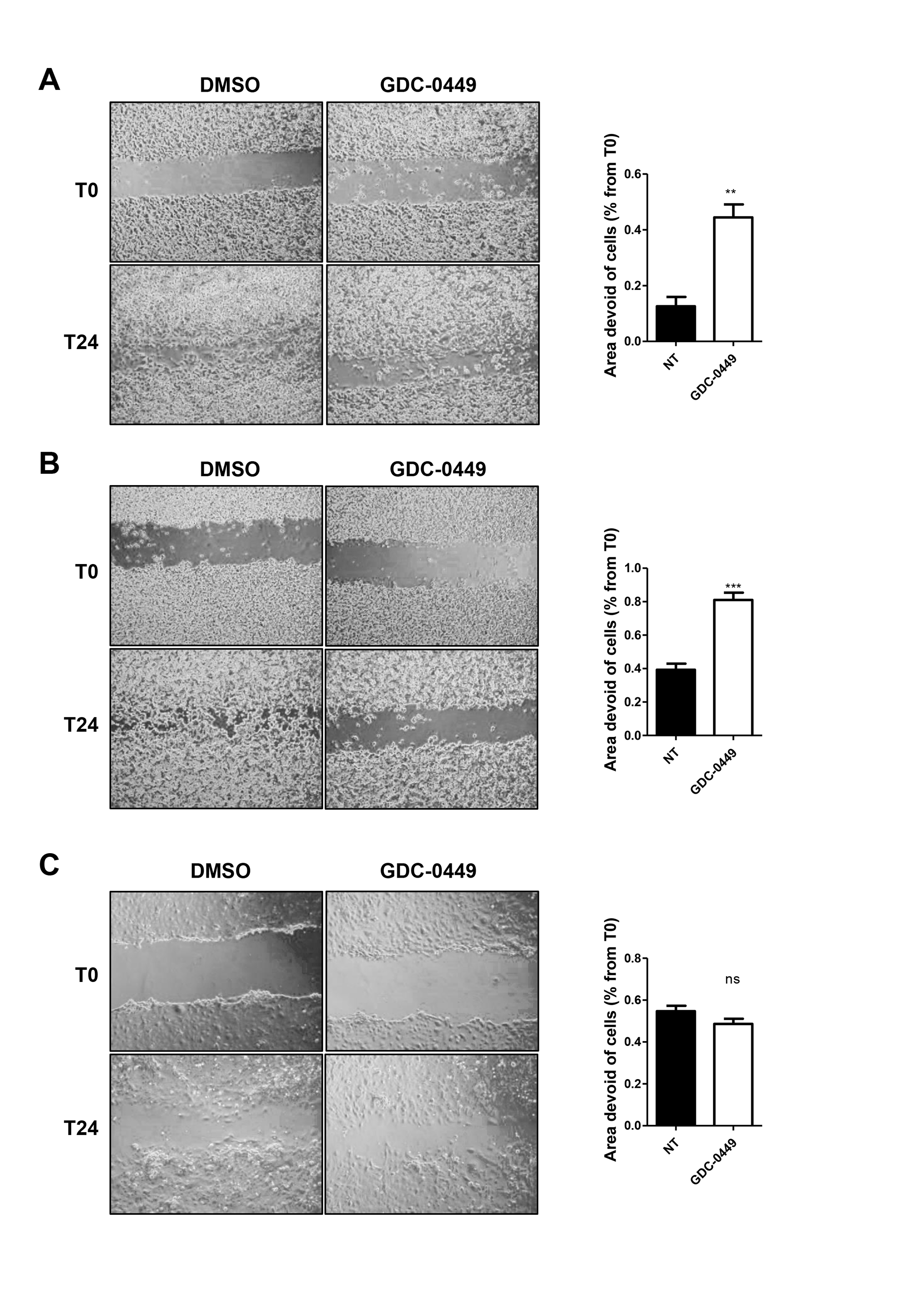

Supplement: Supplementary file 4 [file Image4.TIF]

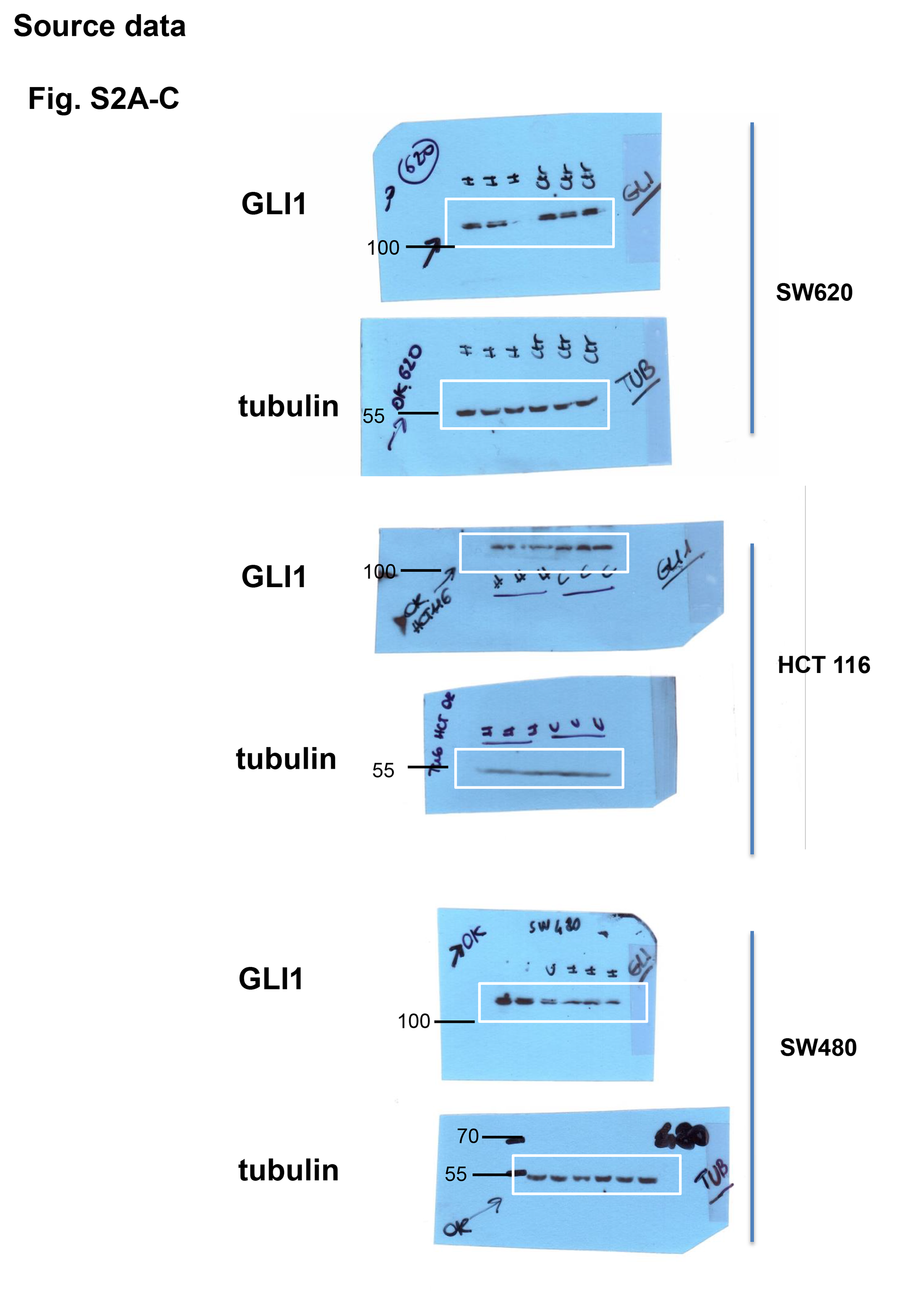

Supplement: Supplementary file 5 [file Image5.TIF]

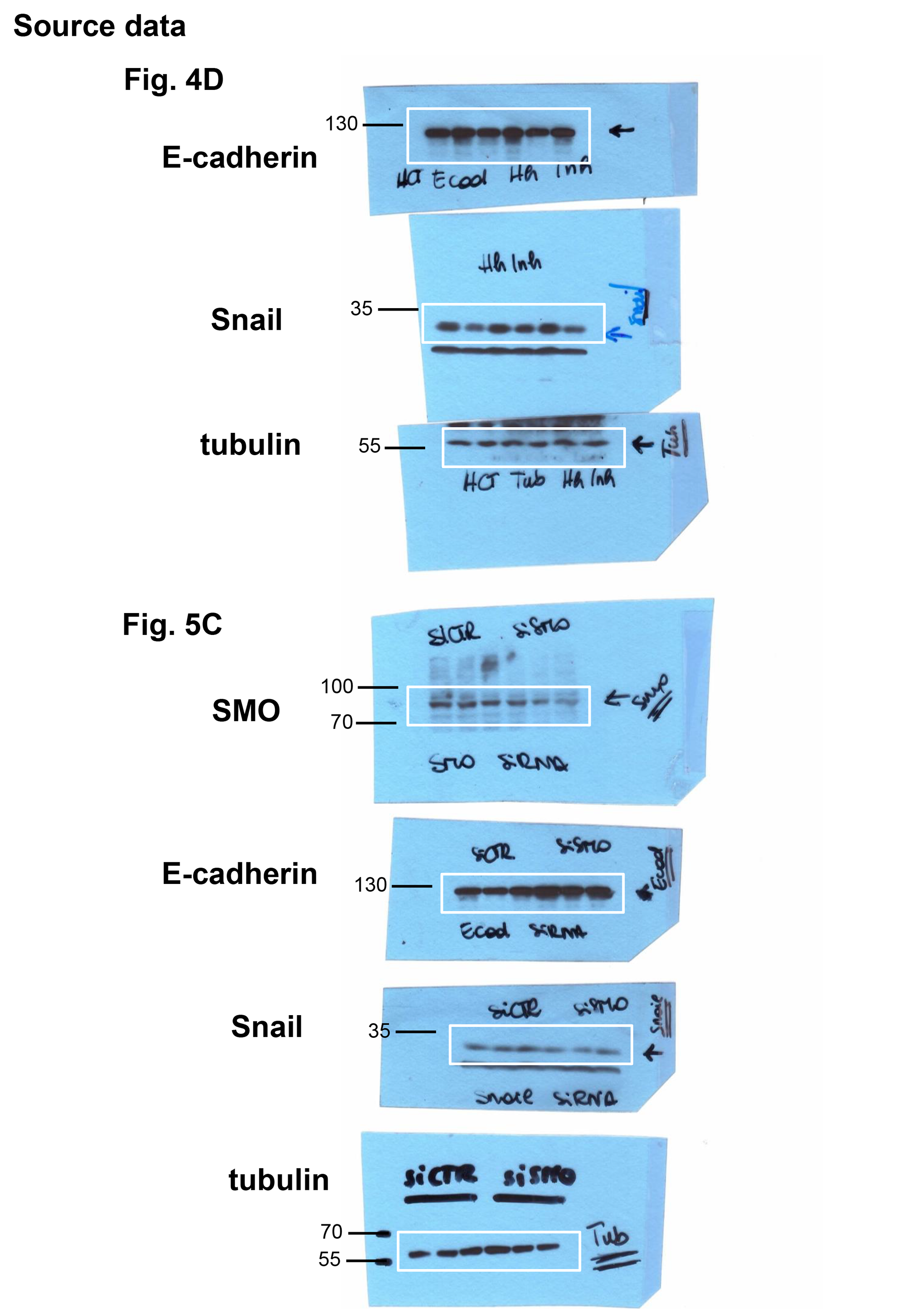

Supplement: Supplementary file 6 [file Image6.TIF]

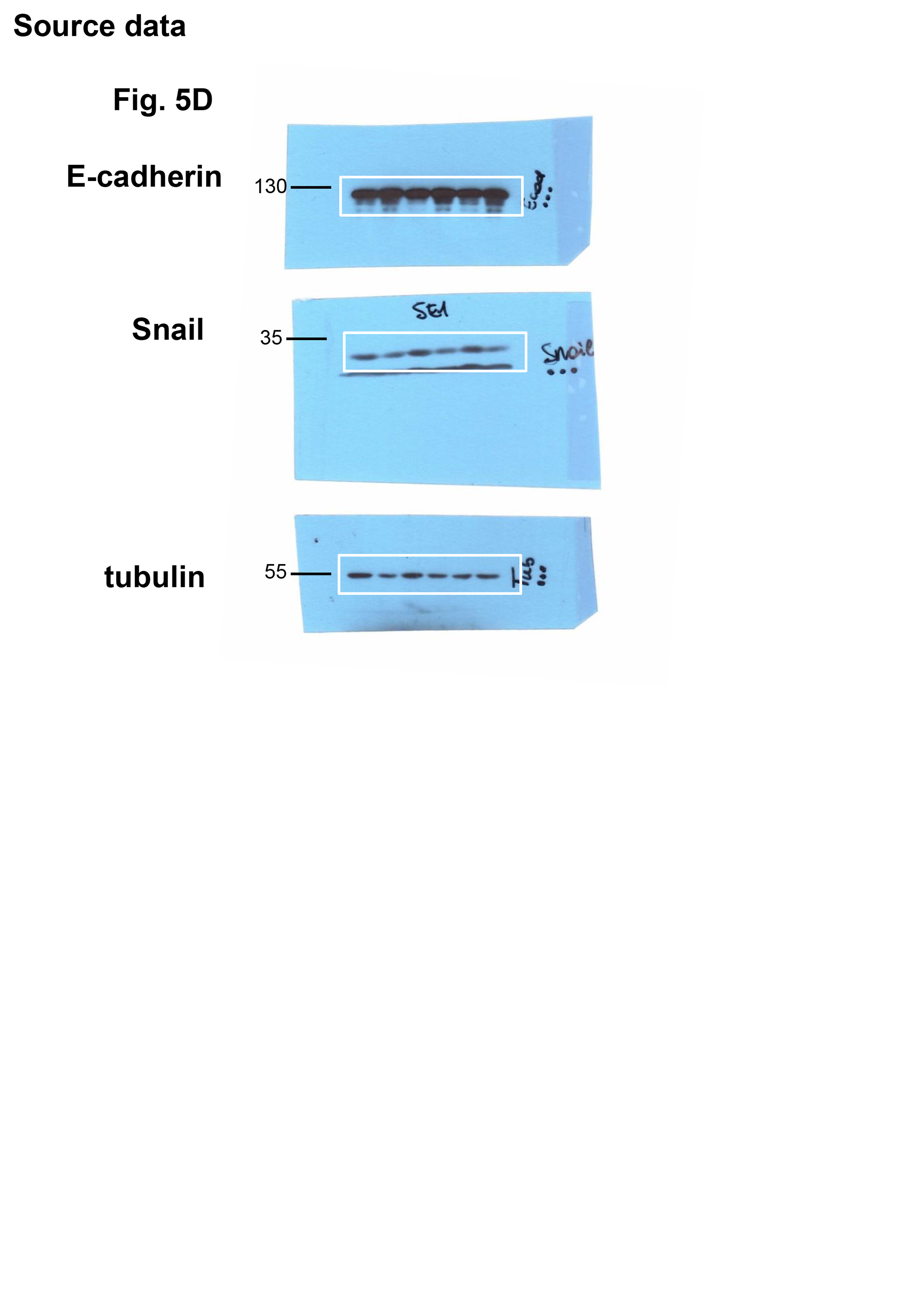

Supplement: Supplementary file 7 [file Image7.TIF]
