# Supplementary material for: SMO Inhibition Modulates Cellular Plasticity and Invasiveness in Colorectal Cancer
Source: Front Pharmacol. 2018 Feb 2;8:956. doi: 10.3389/fphar.2017.00956 (PMC5801594; doi:10.3389/fphar.2017.00956)
Supplement: Supplementary file 8 [file DataSheet1.doc]

**Supplementary figure legends**

**Figure S1**

*GLI1* increased expression and disease-free survival (DFS) (**A**) and *GLI2* increased expression and overall survival (OS) (**B**) by gene expression microarray from a cohort of 382 CC patients in the cancer genome atlas (TCGA) database. The cutoff was z=1.2.  Survival analysis significance was based on Logrank Test. *P*<0.05 was considered significant.

**Figure S2**

Effect of Hh inhibition on GLI1 protein expression. HCT 116 (**A**), SW480 (**B**) and SW620 (**C**) were treated with DMSO vehicle (NT) or with 1 μM GDC-0449 for 24 h. Expression of GLI1 was performed by western blot on total protein lysates. Tubulin was used as a loading control. Three independent experiments were performed. Quantification of GLI1 protein expression is shown on the right. *P*<0.05 was considered significant.

**Figure S3**

Effect of Hh inhibition on Hh pathway-induced genes. (**A**) *SMO* mRNA expression was evaluated on total RNA by qRT-PCR from HCT 116 cells transfected with either control or specific *SMO*-targeting siRNAs. Bars represent means ± SEM of three independent experiments.

(**B**) MeT5A cells were treated with DMSO vehicle (NT) or with 1μM GDC-0449 for 24 h. Expression of *GLI1, PTCH1, HIP1, MUC5AC* was evaluated on total RNA by qRT-PCR. Bars represent means ± SEM of three independent experiments. (**C**) Effect of Hh inhibition on proliferation-related genes. MeT5A cells were treated with DMSO vehicle (NT) or with 1μM GDC-0449 (Hh inh) for 24 h. Expression of *Cyclin D1* (left) and of *p21* (right) was evaluated on total RNA by qRT-PCR. Bars represent means ± SEM of three independent experiments.

**Figure S4**

Effect of Hh inhibition on cell directed migration. SW480 (**A**), SW620 (**B**), MeT5A (**C**) cells were treated with vehicle (DMSO) or with GDC-0449 (1μM) in culture medium supplemented with 0.5% FCS. A scratch was performed and micrographs were taken 24 h after the scratch. Wound closure rate was quantified (right). Three independent experiments were performed. Bars represent means ± SEM of 3 independent experiment. ***P*<0.01, ****P*<0.001 n.s.: not significant

**Figure S5-S7**

Data source of the western blots shown in this study
